# Clinical analysis and rational evaluation of 25 cases of tigecycline: A cross-sectional study

**DOI:** 10.1097/MD.0000000000045522

**Published:** 2025-10-24

**Authors:** Mengmeng Dou

**Affiliations:** a The First People’s Hospital of Lin’an District of Hangzhou, Hangzhou, China.

**Keywords:** rational use, tigecycline

## Abstract

This study aims to evaluate the appropriateness of tigecycline (TC) use in our hospital, provide evidence-based recommendations for its clinical application in the region, and optimize pharmacological strategies accordingly. A cross-sectional study was conducted to extract data from hospitalized patients who received TC in 2020, following the rules for clinical evaluation and application of carbapenem antibiotics. Demographic data, medication regimens, pathogen profiles, and treatment appropriateness were statistically analyzed. The study included 25 patients treated with TC (aged 45–92 years), primarily admitted to the intensive care unit, neurosurgery, or infectious diseases departments. The median treatment duration ranged from 4 to 15 days. Multidrug-resistant *Klebsiella pneumoniae* and *Acinetobacter baumannii*, along with *Pseudomonas aeruginosa*, were the most frequently isolated pathogens. The primary indications were pulmonary and biliary tract infections, with off-label use constituting the main deviation from clinical guidelines. Enhanced management of TC use among inpatients is essential in our hospital. Multidisciplinary collaboration and strengthened drug resistance surveillance are necessary to ensure the rational use of TC.

## 1. Introduction

Tigecycline (TC) is a novel glycylcycline antibiotic commonly administered via intravenous injection. In 2005, the US Food and Drug Administration approved its use for complicated skin and soft tissue infections, intra-abdominal infections, and other adult indications.^[1]^ It exhibits a higher affinity for bacterial ribosomes than tetracyclines and disrupts protein synthesis by inhibiting peptide chain elongation, thereby exerting bactericidal or bacteriostatic effects.^[2]^ In recent years, the widespread use of tigecycline has been accompanied by increasing multidrug resistance in *Klebsiella pneumoniae* and *Acinetobacter baumannii*, posing challenges for rational clinical use.^[3–5]^ This study conducted a cross-sectional analysis of TC use in our hospital in 2020 to evaluate its clinical effectiveness and rationality.

## 2. Materials and methods

### 2.1. Data source and collection

We accessed the hospital’s internal information system to identify 25 hospitalized patients treated with TC between January and December 2020 as the study population. The study was approved by the Hospital Ethics Committee with an exemption from informed consent. First, patient data related to TC use were extracted from the hospital intranet and compiled into an Excel spreadsheet, including demographic information, disease status, medication details, adverse reactions, and other relevant variables. After reviewing the medical records, a structured questionnaire was completed, and the rationality of TC use was evaluated based on a scoring system.

### 2.2. Criteria for assessing medication rationality

According to the Evaluation Rules for Clinical Application of TC,^[6]^ a 100-point evaluation table with a deduction-based system was used. Scores ranged from 0, indicating complete irrationality, to 100, representing full rationality. One deduction item addressed potential irrational use and was further classified into 2 distinct categories: reasonable and unreasonable, as illustrated in Table 1.

**Table 1:** Evaluation form of carbapenem antibacterial drugs.

The standard source is “Detailed Rules for Clinical Application Evaluation of Carbendazole Antibacterial Agents” (National Health Office Medical Letter (2018) No. 822)
1. Indications (100 points deducted if not met): Severe patients with complex abdominal infections, complex skin and soft tissue infections, and community-acquired pneumonia; Multiple drug-resistant *Acinetobacter baumannii* infections (excluding central nervous system and urinary tract infections); Carbapenem-resistant Enterobacteriaceae infections (excluding central nervous system and urinary tract infections).
2. Variety selection (10 points per item): tigecycline for injection.
3. Usage (10 points each): generally intravenous drip.
4. Dosage (10 points per dose): Tigecycline for injection: First, it is not recommended to treat extensively resistant gram-negative bacterial infections with a single drug; Second, the first dose load is 100 mg, with a maintenance dose of 50 mg q2h; children ≥8 y old: 8–11 y old, 1.2 mg/kg every 12 h, with a maximum dose of 50 mg every 12 h, and 12–17 y old, 50 mg per h; Third, Liver dysfunction: Patients with mild to moderate liver dysfunction (Child Pugh grades A and B) do not need to adjust the dose; patients with severe liver dysfunction (Child Pugh grades C) should adjust the dose to 100 mg for the first dose, and then 25 mg every 12 h; Finally, when treating HAP or VAP, the dosage can be increased and maintained up to 100 mg q12h; Treatment considerations include doubling the dosage for severe infections caused by CRE and carbapenem-resistant *Acinetobacter baumannii* (CRAB).
5. Compatibility (10 points each): Drugs that cannot pass through a common pipeline: amphotericin B, amphotericin B liposome complex, diazepam, omeprazole, and esomeprazole. Drugs that can pass through a common pipeline include the following drugs or diluents: amikacin, dobutamine, dopamine hydrochloride, gentamicin, fluperidol, sodium lactate ringer injection, lidocaine hydrochloride, metoclopramide, morphine, norepinephrine, piperacillin tazobactam, potassium chloride, propofol, ranitidine hydrochloride, theophylline, and tobramycin.
6. Etiology and efficacy evaluation: (20 points) Prior to the use of anti Southern medicine, corresponding pathogen testing was conducted, referring to bacterial culture (including evidence of effective pathogens outside the hospital); During treatment, dynamic laboratory tests such as blood routine, procalcitonin, and bacterial culture should be conducted to evaluate the efficacy.
7. Prescription and consultation of special grade pit bacteria drugs (10 points each): The prescription is issued by a doctor with a senior professional title and must be supported by information technology: Timely consultation with experts in the hospital or outside the hospital for special grade anti Rong drugs, and consultation records are kept; The use beyond the designated level is limited to 24 h and has corresponding medical records; According to the submission regulations of “National Health Office Medical Development (2017) No. 10”, carry out special file registration management; Regular training and assessment are conducted and recorded for physicians who have been granted the right to prescribe special grade antibacterial drugs

## 3. Results

### 3.1. Basic information of patients

Among the 25 patients who received TC for anti-infective treatment, 18 (72%) were male and 7 (28%) were female. The patients’ ages ranged from 45 to 92 years.

### 3.2. Department distribution and infection status

The patients were primarily admitted to the intensive care unit, neurosurgery, and infectious diseases departments. TC was mainly prescribed for pulmonary and biliary tract infections (Table 2).

**Table 2:** Distribution of infection site.

Infection site	Number	Composition ratio
Pulmonary infection	9	36%
Infectious fever	3	12%
Pulmonary infection + Biliary fever	2	8%
Other (urinary tract infection, intestinal infection, abdominal fever, vertebral infection, respiratory syncytial virus infection)	11	44%
Total	25	100%

### 3.3. Pathogenic examination

All patients underwent pathogen testing, with a 100% detection rate. Samples included sputum, blood, urine, and feces. A total of 32 pathogens were identified, as detailed in Table 3. The predominant pathogens were multidrug-resistant *K. pneumoniae*, as shown in Table 3.

**Table 3:** Distribution of pathogenic bacteria.

Proportion of pathogenic bacteria cases	Number of cases	Composition ratio
*Klebsiella pneumoniae* subsp. Pneumoniae MDRO	13	48.15%
*Acinetobacter baumannii* MDRO	8	25.00%
*Pseudomonas aeruginosa* CR-PA	3	9.38%
Other (*Trichosporon asahii, Enterobacter cloacae*, Citrobacter Coetzer, *Escherichia coli, Alcaligenes faecalis*)	1	15.63%
Total	25	100.00%

MDRO = multidrug-resistant organism.

### 3.4. Medication situation

#### 3.4.1. Usage, dosage, and course of administration of TC

TC was administered intravenously to all patients. During treatment, 18 patients received a loading dose of 100 mg followed by 50 mg every 12 hours, while the other 7 patients received 50 mg as both the initial and maintenance doses (Table 4). A total of 18 patients (72%) received TC for 5 to 14 days, consistent with the recommended duration in the prescribing information. Seven patients (28%) were treated for 4 days or less, and no patients received treatment for more than 14 days (Table 5).

**Table 4:** Distribution of tigecycline usage and dosage.

Usage and dosage	Number of cases
Immediately after the first dose of 100 mg, 50 mg q12h	18
Immediately after the first dose of 50 mg, 50 mg q12h	7
Total	25

**Table 5:** Treatment course of tigecycline.

Number of courses (d)	Number	Percentage(%)
≤4	7	28%
5–14	18	72%
Total	25	100%

#### 3.4.2. Combination and rational use of drugs

Of the 25 patients, 13 were treated with TC monotherapy, while the remaining 12 received combination antibiotic therapy. The most commonly used concomitant antibiotics were imipenem (20%) and cefoperazone/sulbactam (SCF, 12%). Fifteen patients showed a marked antibacterial response (Table 5). Based on the scoring criteria, 1 case (4%) involved non-indicated use, while 96% of the treatments were deemed rational, as shown in Tables 6 and 7.

**Table 6:** Combined use of tigecycline.

CM	Specific categories	Number of cases	Composition ratio
Hydrocarbon enzymes	Imipenem;meropenem	5	45.45%
β-lactam	Cefoperazone sulbactam sodium	3	27.27%
Sulfonamides	Compound sulfamethoxazole	2	18.18%
Antifungal drugs	Fluconazole	1	9.09%
Total	-	11	100.00%

“-” indicates that it does not exist.

CM = concomitant medication.

**Table 7:** Reasonableness judgment of the use of tigecycline.

Category	Reasonable	Unreasonable
Indication	24	1
Medicine regimen	25	0
Etiology and treatment regimen	25	0
Special grade antibacterial drugs and consultations	25	0

## 4. Discussion

TC is clinically indicated for the treatment of complicated intra-abdominal infections, complicated skin and soft tissue infections, and community-acquired pneumonia in adults.^[6]^ A cross-sectional analysis was conducted on TC use among hospitalized patients in 2020. The results showed that TC was used as an anti-infective agent, with a treatment efficacy rate of 96%, appropriate utilization in 96% of cases, and an adverse event rate of 4%.

Studies have shown that TC, when combined with imipenem and cilastatin sodium, is effective for treating acute pancreatitis complicated by abdominal infections. This combination therapy can rapidly relieve clinical symptoms, improve clinical outcomes, and demonstrate a favorable safety profile.^[7]^ Furthermore, TC combined with SCF in patients with severe infections has been shown to enhance coagulation function, reduce inflammatory markers, and decrease the incidence of adverse events.^[8]^ In patients with pulmonary infections caused by carbapenem-resistant *A. baumannii*, TC combined with SCF has shown superior efficacy compared with carbapenem-based regimens.^[9]^

In this study, a total of 32 pathogenic bacteria were isolated. Among patients with multidrug-resistant organism infections, 65.6% were due to gram-negative bacteria, with *K. pneumoniae* and *A. baumannii* accounting for 40.6% and 25% of cases, respectively. Therapeutic outcomes may have been influenced by various factors, including sample size, treatment duration, and underlying clinical complexity. Given previous warnings regarding TC use, clinicians should closely monitor patients during treatment and adopt effective antimicrobial stewardship strategies.^[9–13]^

In conclusion, TC demonstrates strong therapeutic efficacy, and its clinical use is steadily increasing. Although TC use in our hospital was largely rational, management protocols still require improvement. Hospitals are encouraged to provide comprehensive and ongoing training on rational antibiotic use, particularly in general hospitals. Such training should focus on specific antimicrobials, regulate off-label use, and promote rational prescribing to ensure patient safety. Emphasizing the critical role of clinical pharmacists may help mitigate antibiotic resistance due to misuse and improve the overall quality of clinical medication practices.

## Acknowledgments

The author thanks all of the subjects who participated in this study.

## Author contributions

**Conceptualization:** Mengmeng Dou.

**Data curation:** Mengmeng Dou.

**Formal analysis:** Mengmeng Dou.

**Supervision:** Mengmeng Dou.

**Writing – original draft:** Mengmeng Dou.

**Writing – review & editing:** Mengmeng Dou.
